# Epithelial membrane protein 2 (EMP2) regulates hypoxia-induced angiogenesis in the adult retinal pigment epithelial cell lines

**DOI:** 10.1038/s41598-022-22696-x

**Published:** 2022-11-12

**Authors:** Michel Sun, Nina Cherian, Lucia Liu, Ann M. Chan, Brian Aguirre, Alison Chu, Jason Strawbridge, Esther S. Kim, Meng-Chin Lin, Irena Tsui, Lynn K. Gordon, Madhuri Wadehra

**Affiliations:** 1 UCLA Stein Eye Institute and the Department of Ophthalmology, David Geffen School of Medicine at UCLA, Los Angeles, CA USA; 2 Division of Neonatology and Developmental Biology, Department of Pediatrics, David Geffen School of Medicine at UCLA, Los Angeles, CA USA; 3 Department of Pathology Lab Medicine, 4525 MacDonald Research Laboratories, David Geffen School of Medicine at UCLA, Los Angeles, CA 90095 USA; 4 Jonsson Comprehensive Cancer Center, David Geffen School of Medicine at UCLA, Los Angeles, CA USA

**Keywords:** Retinal diseases, Stress signalling

## Abstract

Pathologic retinal neovascularization is a potentially blinding consequence seen in many common diseases including diabetic retinopathy, retinopathy of prematurity, and retinal vaso-occlusive diseases. This study investigates epithelial membrane protein 2 (EMP2) and its role as a possible modulator of angiogenesis in human retinal pigment epithelium (RPE) under hypoxic conditions. To study its effects, the RPE cell line ARPE-19 was genetically modified to either overexpress EMP2 or knock down its levels, and RNA sequencing and western blot analysis was performed to confirm the changes in expression at the RNA and protein level, respectively. Protein expression was evaluated under both normoxic conditions or hypoxic stress. Capillary tube formation assays with human umbilical vein endothelial cells (HUVEC) were used to evaluate functional responses. EMP2 expression was found to positively correlate with expression of pro-angiogenic factors HIF1α and VEGF at both mRNA and protein levels under hypoxic conditions. Mechanistically, EMP2 stabilized HIF1α expression through downregulation of von Hippel Lindau protein (pVHL). EMP2 mediated changes in ARPE-19 cells were also found to alter the secretion of a paracrine factor(s) in conditioned media that can regulate HUVEC migration and capillary tube formation in in vitro functional angiogenesis assays. This study identifies EMP2 as a potential mediator of angiogenesis in a human RPE cell line. EMP2 levels positively correlate with pro-angiogenic mediators HIF1α and VEGF, and mechanistically, EMP2 regulates HIF1α through downregulation of pVHL. This study supports further investigation of EMP2 as a promising novel target for therapeutic treatment of pathologic neovascularization in the retina.

## Introduction

Neovascularization (NV), the growth of new vasculature structures from existing ones, is a regulated process involved in the repair of various bodily tissues. Pathologic NV in the eye, however, is associated with numerous pathologies, that may result in severe visual impairment^1^. Within the eye, pathologic NV is most commonly observed in the cornea and retina, in diseases such as diabetic retinopathy, retinopathy of prematurity, age-related macular degeneration, and corneal neovascularization^2^.

Vascular endothelial growth factor (VEGF) is an important regulator of normal vascular development throughout the body^3^. However, up-regulation of pro-angiogenic factors like VEGF is also responsible for a variety of abnormal neovascular pathologies including pathologic ocular NV^4^. Ongoing research focuses on characterizing modulation of VEGF by regulatory proteins including hypoxia inducible factor 1-alpha (HIF1α), a protein identified to be an upstream regulator of VEGF and angiogenesis in experimental models of hypoxia-mediated neovascularization^5,6^. Inhibiting these pro-angiogenic factors is proven to be an effective strategy in therapeutic intervention, with intraocular anti-VEGF treatments demonstrating large reductions of NV in diabetic retinopathy, retinopathy of prematurity, and other ocular vascular pathologies^7,8^. However, these therapies are not risk-free, and there may be loss of therapeutic efficacy over time^9,10^. Accordingly, identifying other mechanisms that control pathologic NV could lead to much needed new therapeutic options.

Epithelial membrane protein 2 (EMP2) is expressed in multiple ocular tissues including the corneal epithelium and the retinal pigment epithelium^11^. We previously reported that EMP2 expression positively correlates with VEGF levels in the RPE-like cell line, ARPE-19^12^. Recently, we described a role for EMP2 in an alkali burn model of corneal neovascularization. Blocking EMP2 in a murine in vivo model of corneal neovascularization resulted in a significant decrease of NV; additionally, anti-EMP2 treatment subsequently decreased VEGF levels in animal models and in vitro in corneal endothelial cells^13^. In addition, in a murine model of oxygen-induced retinopathy (OIR), EMP2 KO mice demonstrated attenuated NV via downregulation of HIF1α and VEGF expression^8^. Outside of the eye, we have observed a correlation between levels of EMP2 expression and pathologic angiogenesis in a variety of tumor models in vitro including breast cancers^14^, endometrial cancers^15,16^, and glioblastoma^17–19^. However, the effect of hypoxia on EMP2 expression in RPE cells has not been investigated. In this paper, we demonstrate that expression of epithelial membrane protein 2 (EMP2) regulates von Hippel Lindau tumor suppressor protein (pVHL), leading to modulation of HIF1α, and subsequent neoangiogenic induction by ARPE-19 cells.

## Results

### EMP2 is highly expressed within RPE and RPE cell lines

**Figure 1:**
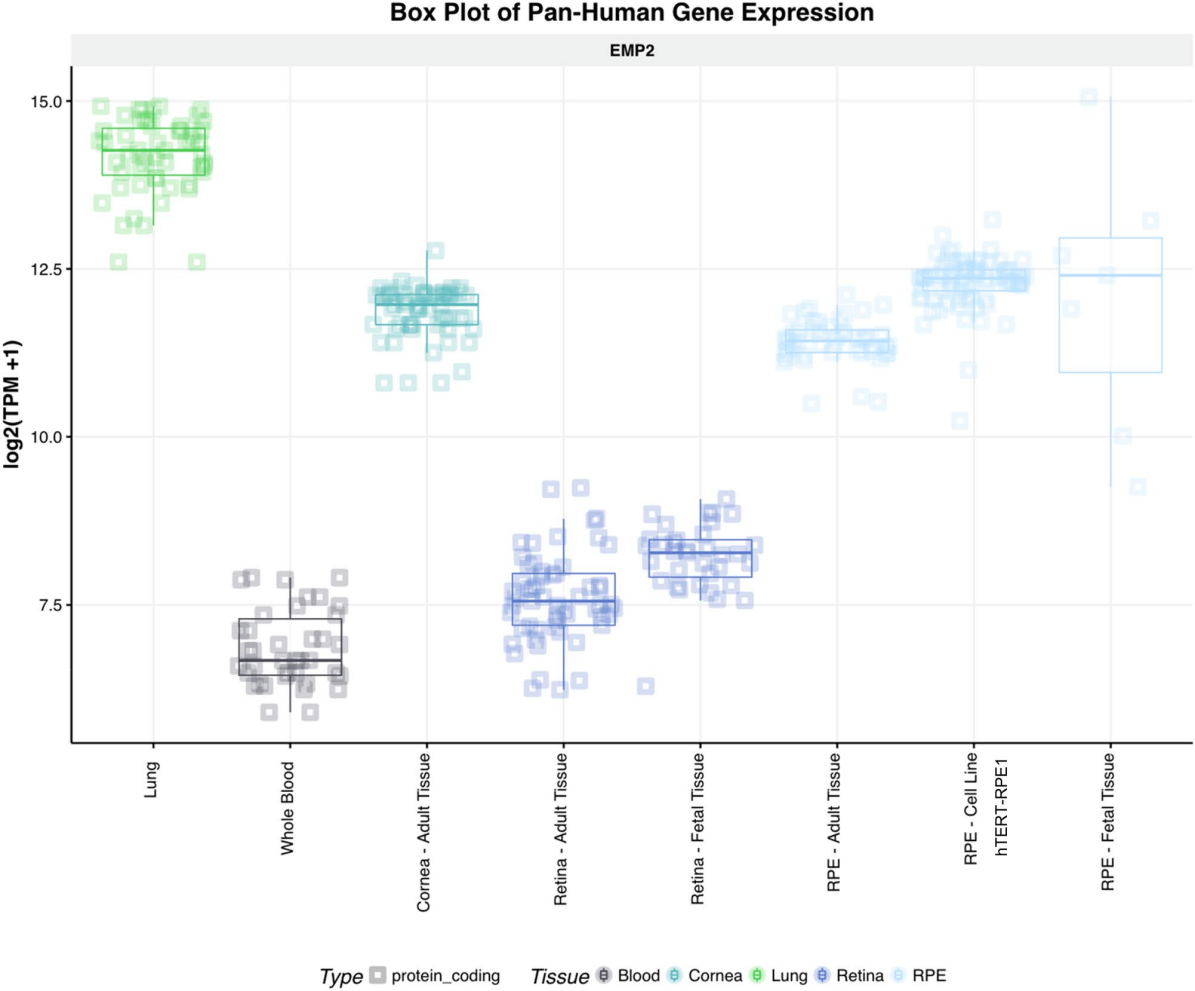
Differential expression of EMP2 in ocular tissues. Visualization generated using the NEI EyeIntegration web resource (https://eyeIntegration.nei.nih.gov), queried for differential expression of EMP2 in specific eye tissues of interest including adult cornea, adult retina, fetal retina, adult RPE, fetal RPE, and RPE cell line. Expression within these ocular sites was compared to EMP2 in the lung and whole blood, sites confirmed to have high and low expression of the transcript, respectively^17^. RPE cell line used is the hTERT-RPE1 line. Data obtained from EyeIntegration version 1.05 using the Gene 2019 dataset.

To examine the expression profile of EMP2, we investigated the NEI’s eyeIntegration database, a large collection of RNA-seq gene expression datasets focusing on profiling specific ocular tissues^20^. Consistent with our published work^11^, EMP2 was highly expressed in lung, cornea, and RPE, with low expression in neurosensory retina under normal conditions (Fig. 1). Interestingly, EMP2 expression was higher in fetal retina than adult retina and also higher in fetal RPE than adult RPE, suggesting it may be important during early eye development. EMP2 was also highly expressed in the hTERT-RPE1 cell line used in this gene expression database, with expression in cell lines higher than expression in adult RPE and more consistent with expression levels of fetal RPE.

### ARPE-19 cells modified to change EMP2 expression levels still express traditional RPE markers

**Figure 2:**
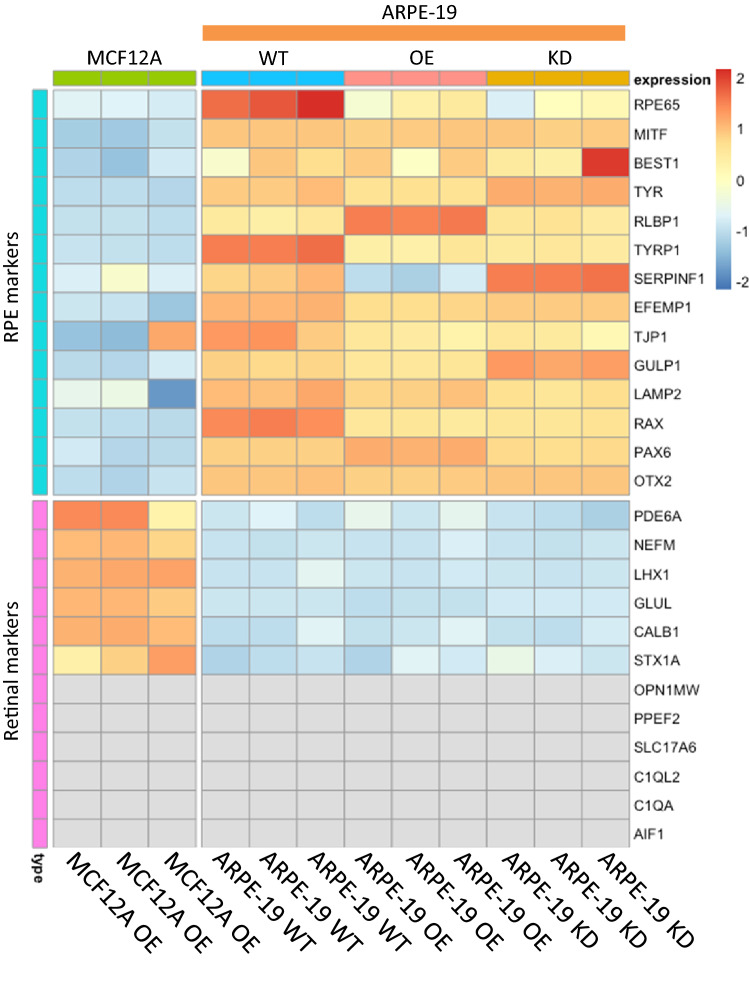
RNA sequencing of ARPE-19 cells for RPE and retinal markers. Wild type (WT), EMP2 overexpressing (OE), and EMP2 knock down (KD) ARPE-19 cells were queried for differential expression of classical RPE markers and retinal markers. A control cell line, MCF12A (immortalized breast cancer cell line) was analyzed in parallel, and results are presented as a heatmap generated in R studio using pheatmap, version 1.0.12 (https://CRAN.R-project.org/package=pheatmap.) High expression of RPE markers and low expression of retinal markers was observed in ARPE-19 cells, regardless of EMP2 expression. Both MCF12A and ARPE-19 cells showed low expression of most retinal markers (gray indicating zero transcripts identified). All samples were performed in triplicate.

We used the immortalized ARPE-19 RPE-like cell line, which has a high baseline expression of EMP2 mRNA and protein, for the reported studies. ARPE-19 cells were genetically modified to either overexpress EMP2 (OE) or knock down EMP2 (KD), and expression was evaluated using RNA sequencing. To confirm the appropriateness of the model, wild type (WT) ARPE-19 cells were first analyzed for RPE specific markers and its profile compared to MCF12A mammary epithelial cell lines that overexpress EMP2. WT ARPE-19 cells express high levels of specific RPE markers, including RPE65, MITF, BEST1, TYR, RLBP1, TYRP1, SERPINF1, EFEMP1, TJP1, GULP1, LAMP2, RAX, PAX6, and OTX2, consistent with a classical RPE phenotype (Fig. 2). In contrast, MCF-12A cells demonstrated low or undetectable levels of all RPE markers.

The expression profile of the EMP2 modified cell lines were next assessed. Both the EMP2 OE and KD cell lines demonstrated similar expression to WT of all traditional RPE markers, suggesting genetic modification of EMP2 did not result in loss of typical RPE markers. The RPE cell lines all demonstrated comparatively low expression of neuroretina specific markers such as PDE6A, NEFM, LHX1, GLUL, CALB1, STX1A, and no detectable expression of the neuroretina markers OPN1MW, PPEF2, SLC17A6, C1QL2, C1QA, and AIF1 (Fig. 2). These data support the RPE lineage of the ARPE-19 cell line, and demonstrate no significant alterations in RPE lineage with changes in EMP2 expression.

### ARPE-19 cells modified to change EMP2 expression levels differentially express genes related to angiogenesis and vascular development

**Figure 3:**
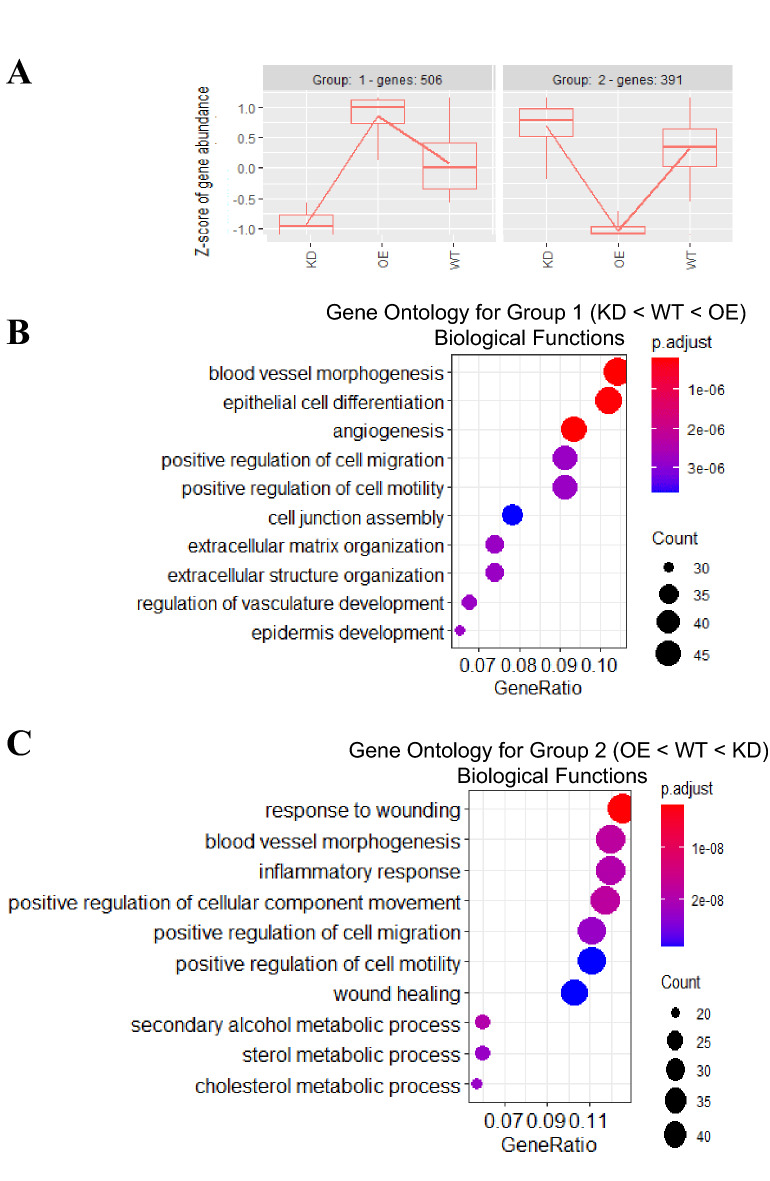
Transcriptome patterns and properties of genes impacted by EMP2 modification. Changes in gene expression across wild type (WT), EMP2 overexpressing (OE), and EMP2 knock down (KD) ARPE-19 cells were queried by the likelihood ratio test. (**A**) Hierarchical clustering analysis of the top 1000 significant genes (FDR < 0.01) by the *degPatterns* tool within the DEGreport R package (version 1.26.0) reveals four distinct expression profile groups. (**B**, **C**) Enriched biological property gene ontology (GO) terms for group 1 and group 2 cluster genes, respectively. The top 10 GO terms ordered by gene ratio (number of GO term-related genes divided by total number of significant genes) identify angiogenesis and vasculature-related terms as significantly enriched for group 1 genes. Dot color represents the Benjamini–Hochberg adjusted *p* value for each enriched GO term. All samples were performed in triplicate.

To further characterize genes with higher or lower abundance that correlated with EMP2 levels, we performed gene ontology (GO) term over-representation analysis. Over 8000 genes were differentially expressed by alterations in EMP2 (adjusted *p* < 0.01), with the top 1000 genes being selected for clustering and enrichment analysis. Three conditions (KD, WT, and OE) were used for hierarchical clustering, with four groups of genes observed that clustered according to EMP2 levels (Fig. 3A). Group 1 enriched for genes associated with high EMP2 levels, and 506 genes positively correlated with EMP2 levels. In contrast, group 2 revealed 391 genes with reciprocal expression. Several GO terms involved with angiogenesis and blood vessel morphogenesis were enriched by EMP2 (Fig. 3B) while low EMP2 levels altered processes associated with metabolism, cellular immunity, and motility (Fig. 3C). A summary of genes present within these GO terms, enriched or reduced by EMP2, are presented in Tables 1 and 2.

**Table 1:** Gene Set Enrichment Analysis was performed on differentially expressed transcripts in ARPE-19 cells with modified EMP2 levels, with the top 10 Go ontology terms from Group 1 (KD < WT < EMP2) shown.

Description	Count	Genes
Blood vessel morphogenesis	48	ADGRG1/ADTRP/ANGPTL4/ANXA3/B4GALT1/BMPER/CCN2/COL4A1/COL4A2/COL4A3/CTSH/CXCL8/EFNB2/EMC10/EMP2/ENPP2/EPHA2/HDAC7/HEG1/HES1/HIF1A/IL18/ITGA5/ITGAV/JUNB/JUP/LOX/LOXL2/MCAM/MMP2/MYH9/MYOCD/NPPB/PARVA/PLK2/PLXND1/PTGIS/RHOB/SERPINE1/SFRP1/SHB/SPHK1/SULF1/TGFB2/TGFBI/THBS1/TNFRSF12A/UNC5B
Angiogenesis	43	ADGRG1/ADTRP/ANGPTL4/ANXA3/B4GALT1/BMPER/CCN2/COL4A1/COL4A2/COL4A3/CTSH/CXCL8/EFNB2/EMC10/EMP2/ENPP2/EPHA2/HDAC7/HIF1A/IL18/ITGA5/ITGAV/JUP/LOXL2/MCAM/MMP2/MYH9/NPPB/PARVA/PLK2/PLXND1/PTGIS/RHOB/SERPINE1/SFRP1/SHB/SPHK1/SULF1/TGFB2/TGFBI/THBS1/TNFRSF12A/UNC5B
Epithelial cell differentiation	47	ACADVL/ACTA2/AFDN/AJAP1/AKR1C3/ARHGEF26/B4GALT1/CCND1/CDKN1A/CLDN1/COL4A1/CYP26B1/DSC2/DSG2/ELF3/EPHA2/ERBB4/EZR/HEG1/HES1/HIF1A/ICAM1/IVL/JUP/KRT13/KRT18/KRT33B/KRT7/KRT80/KRT86/LBH/LIF/MAF/MYO7A/NRG1/PALLD/PAX6/PODXL/RARA/SERPINE1/SFRP4/SIPA1L3/SRC/TGM1/TP63/TPRN/TRIOBP
Positive regulation of cell migration	42	ANXA3/BDKRB1/CLDN1/CSF1R/CTSH/CXCL8/DOCK5/EDN2/EMC10/ENPP2/FAM83H/FOXP1/GPER1/HDAC7/HIF1A/ICAM1/IGF1R/IGFBP5/ITGA5/ITGAV/MCAM/MYO1C/NEDD9/NOX4/P2RY6/PDGFD/PIK3R1/PLK2/PODXL/RAC2/RHOB/SEMA3B/SEMA7A/SERPINE1/SH3RF2/SPHK1/SRC/SSH1/TGFB2/THBS1/VSIR/ZNF268
Regulation of vasculature development	31	ANGPTL4/ANXA3/BMPER/COL4A2/COL4A3/CTSH/CXCL8/EFNB2/EMC10/EMP2/ENPP2/EPHA2/GPER1/HDAC7/HIF1A/ITGA5/JUP/MYOCD/NPPB/PDGFD/PLK2/PLXND1/PTGIS/RHOB/SERPINE1/SFRP1/SPHK1/SULF1/TGFB2/THBS1/TNFRSF12A
Epidermis development	30	AKR1C3/CCN2/CYP26B1/DSC2/DSG2/EPHA2/FOXQ1/HES1/IGFBP5/IVL/JUP/KRT13/KRT18/KRT33B/KRT7/KRT80/KRT86/LAMB3/LGR4/MYO7A/PALLD/PAX6/PLOD1/SFRP4/TGFB2/TGM1/TNFRSF19/TP63/TPRN/TRIOBP
Positive regulation of cell motility	42	ANXA3/BDKRB1/CLDN1/CSF1R/CTSH/CXCL8/DOCK5/EDN2/EMC10/ENPP2/FAM83H/FOXP1/GPER1/HDAC7/HIF1A/ICAM1/IGF1R/IGFBP5/ITGA5/ITGAV/MCAM/MYO1C/NEDD9/NOX4/P2RY6/PDGFD/PIK3R1/PLK2/PODXL/RAC2/RHOB/SEMA3B/SEMA7A/SERPINE1/SH3RF2/SPHK1/SRC/SSH1/TGFB2/THBS1/VSIR/ZNF268
Extracellular matrix organization	34	ADAMTS2/ADAMTS5/ADAMTS7/ADTRP/B4GALT1/CCN2/COL4A1/COL4A2/COL4A3/COL5A1/COL9A2/DDR1/ELF3/FLRT2/ICAM1/ITGA5/ITGA7/ITGAV/ITGB5/LAMB3/LOX/LOXL2/LTBP3/MELTF/MMP2/MMP24/PHLDB1/RIC1/SERPINE1/SULF1/SULF2/TGFB2/TGFBI/THBS1
Extracellular structure organization	34	ADAMTS2/ADAMTS5/ADAMTS7/ADTRP/B4GALT1/CCN2/COL4A1/COL4A2/COL4A3/COL5A1/COL9A2/DDR1/ELF3/FLRT2/ICAM1/ITGA5/ITGA7/ITGAV/ITGB5/LAMB3/LOX/LOXL2/LTBP3/MELTF/MMP2/MMP24/PHLDB1/RIC1/SERPINE1/SULF1/SULF2/TGFB2/TGFBI/THBS1
Cell junction assembly	36	ACTN1/AFDN/AMIGO2/ARVCF/ASIC2/BDNF/CDH10/CDH11/CLDN1/CLSTN2/CORO1C/EFNB2/EPHA2/ERBB4/FLRT2/FLRT3/FMN1/GPC4/HDAC7/HEG1/ITGA5/JUP/LAMB3/LIMCH1/LSR/MYO1C/NLGN4X/NRG1/OXTR/PLXND1/PTPRJ/SFRP1/SRC/THBS1/TNS1/UGT8

**Table 2:** Gene Set Enrichment Analysis was performed on differentially expressed transcripts in ARPE-19 cells with modified EMP2 levels, with the top 10 Go ontology terms from Group 2 (EMP2 < WT < KD) shown.

Description	Count	Genes
Response to wounding	44	ABHD2/ANO6/ANXA6/APOE/CAV1/CD109/CD36/CSRP1/DOCK8/DPYSL3/ETS1/F2R/F2RL2/F3/FBLN1/GAS6/GLI3/GNA12/ITGB1/ITPK1/KCNK2/MDK/MYLK/MYOF/NREP/NRP1/PLAT/PLAU/PPARD/PRCP/PRKAR2B/PTPRF/PTPRS/RAB27A/SDC1/SERPINA1/SH2B3/TFPI/TGFBR2/TIMP1/TLR4/TNFAIP3/VANGL2/WNT5B
Blood vessel morphogenesis	42	ADGRA2/ANGPTL2/APOE/ATP2B4/C3/CAV1/CCBE1/COL8A2/CREB3L1/CYP1B1/ECM1/EGFL7/EPAS1/ETS1/F3/FGFR1/FMNL3/FZD8/GPNMB/HMGA2/ITGB1/LDLR/LEF1/LRP5/MDK/MMP14/MMP19/MYLK/NRP1/PRCP/PRRX1/PTPRB/ROBO4/SERPINF1/SHC1/SMO/SPRY2/ STAT3/TGFBR2/TIE1/TNFAIP3/ZMIZ1
Positive regulation of cellular component movement	41	ADGRA2/ANO6/ARHGEF2/CAV1/CCBE1/CCL7/CD274/CEMIP/CMKLR1/CSF1/DAB2/DOCK8/ETS1/F2R/F3/FGFR1/GAS6/GPNMB/IL1R1/ITGA6/ITGB1/LAMB1/LEF1/LPAR1/MAP2/MDK/MMP14/MMP9/MYLK/NRP1/PLAU/PREX1/SEMA3F/SEMA6B/SMO/SOD2/SPRY2/STAT3/ STMN1/TGFBR2/WNT5B
Inflammatory response	42	ANO6/APOE/C3/C4B/CAMK4/CCL7/CD36/CLU/CMKLR1/CSF1/ECM1/ETS1/F2R/F3/HRH1/IFI16/IL1R1/IL1RAP/IL4R/IRAK2/LDLR/MAPT/MDK/MGLL/MMP9/MVK/NAMPT/OSMR/PPARD/PRCP/PTX3/PXK/S1PR3/SDC1/SERPINA1/SERPINF1/SMO/STAT3/TIMP1/TLR4/TNFAIP3/TNFRSF11A
Secondary alcohol metabolic process	21	ABCA1/ACAT2/APOE/DGAT2/DHCR7/ELOVL6/FDFT1/FDPS/FGFR1/IDI1/LDLR/LRP5/LSS/MSMO1/MVD/MVK/NPC1/PPARD/SCD/SQLE/ SREBF2
Positive regulation of cell migration	39	ADGRA2/ANO6/ARHGEF2/CAV1/CCBE1/CCL7/CD274/CEMIP/CMKLR1/CSF1/DAB2/DOCK8/ETS1/F2R/F3/FGFR1/GAS6/GPNMB/IL1R1/ITGA6/ITGB1/LAMB1/LEF1/LPAR1/MDK/MMP14/MMP9/MYLK/NRP1/PLAU/PREX1/SEMA3F/SEMA6B/SMO/SOD2/SPRY2/STAT3/TGFBR2/WNT5B
Cholesterol metabolic process	20	ABCA1/ACAT2/APOE/DGAT2/DHCR7/ELOVL6/FDFT1/FDPS/IDI1/LDLR/LRP5/LSS/MSMO1/MVD/MVK/NPC1/PPARD/SCD/SQLE/SREBF2
Sterol metabolic process	21	ABCA1/ACAT2/APOE/CYP1B1/DGAT2/DHCR7/ELOVL6/FDFT1/FDPS/IDI1/LDLR/LRP5/LSS/MSMO1/MVD/MVK/NPC1/PPARD/SCD/SQLE/ SREBF2
Wound healing	36	ANO6/ANXA6/APOE/CAV1/CD109/CD36/CSRP1/DOCK8/ETS1/F2R/F2RL2/F3/FBLN1/GAS6/GLI3/GNA12/ITGB1/ITPK1/MYLK/MYOF/PLAT/PLAU/PPARD/PRCP/PRKAR2B/RAB27A/SDC1/SERPINA1/SH2B3/ TFPI/TGFBR2/TIMP1/TLR4/TNFAIP3/VANGL2/WNT5B
Positive regulation of cell motility	39	ADGRA2/ANO6/ARHGEF2/CAV1/CCBE1/CCL7/CD274/CEMIP/CMKLR1/CSF1/DAB2/DOCK8/ETS1/F2R/F3/FGFR1/GAS6/GPNMB/IL1R1/ITGA6/ITGB1/LAMB1/LEF1/LPAR1/MDK/MMP14/MMP9/MYLK/NRP1/PLAU/PREX1/SEMA3F/SEMA6B/SMO/SOD2/SPRY2/STAT3/TGFBR2/WNT5B

### EMP2 expression modulates HIF1α and VEGF mRNA expression

**Figure 4:**
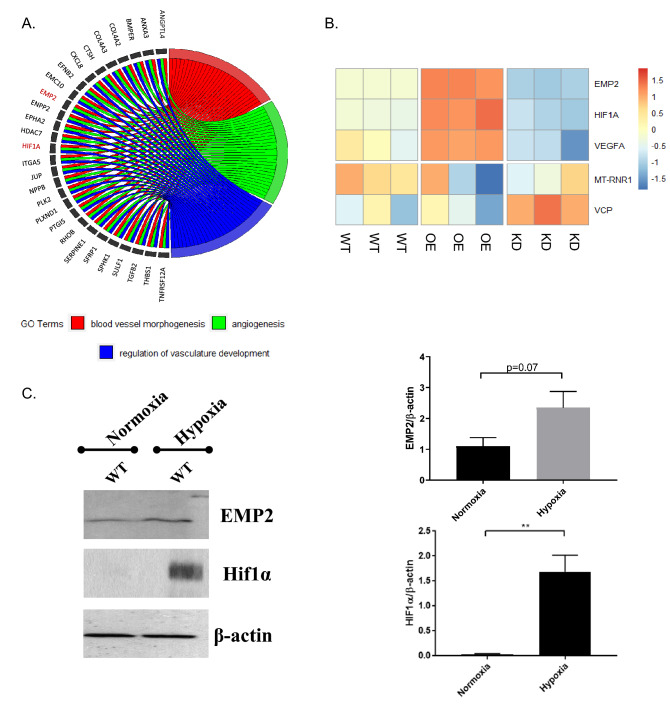
EMP2 regulates gene sets related to blood vessel morphogenesis. (**A**) Intersection of genes involved in neoangiogenesis and its related pathways depicted using a chord diagram. The chord diagram was generated in "GOplot", version 1.0.2 (https://CRAN.R-project.org/package=GOplot). (**B**) Heatmap of EMP2, HIF1α, and VEGFA expression in ARPE-19 cells with modified EMP2 levels. Successful EMP2 transcript alteration was confirmed for wild type (WT), EMP2 overexpressing (OE), and EMP2 knock down (KD) ARPE-19 cells. HIF1α and VEGFA expression correlates with EMP2 expression, with increased transcript levels in OE and decreased transcript levels in KD. The housekeeping genes MT-RNR1 (16S rRNA) and VCP were used as controls for normalization. All samples were performed in triplicate. (**C**) HIF1α expression is induced in ARPE-19 WT under hypoxic conditions. Analysis of HIF1α and EMP2 protein expression in WT ARPE-19 cells under normoxic conditions and following 2–4 h of hypoxic stress (0.5% O_2_). Bands were visualized using autoradiography. Bar diagrams depict mean values and standard error of the mean. Statistical significance was established using a Student’s *t*-test (unpaired, two-tailed). **, *p* = 0.0076. Original, uncropped blots are presented in Supplementary Fig. S3.

Several gene sets important for angiogenesis, such as those correlated with blood vessel morphogenesis and vascular development, correlate with changes in EMP2 expression. Using a chord diagram to display the inter-relationships between genes involved in vasculogenesis, 27 genes were isolated as being enriched by EMP2 (Fig. 4A). Further interrogation of differentially expressed genes in the OE and KD groups revealed significant changes (FDR < 0.01) in HIF1α and VEGF mRNA expression (Fig. 4B). Both transcripts significantly correlated with EMP2 levels, suggesting EMP2 may play a role in modulating select angiogenic factors in RPE cells. As expected, expression of EMP2 was significantly higher in the overexpressing line and lower in the knock down, confirming effective modulation in this ARPE-19 panel. The mitochondrial gene MT-RNR1 and valosin-containing protein gene VCP, housekeeping genes for gene expression normalization, showed no statistically significant differences between groups.

### EMP2 expression modulates HIF1α, VEGF, and VHL protein expression

Given the effects of EMP2 on HIF1α and VEGF expression, we next determined if EMP2 itself could be regulated by hypoxia (Fig. 4C). As expected, hypoxia increased HIF1α expression in WT ARPE-19 cells, and while an increase was also detected in EMP2 protein levels, this effect was not significant.

We next investigated the effects of EMP2 over- or under- expression during normoxic conditions and in the setting of hypoxic stress. In these experiments, two constructs were compared to verify the effects of the EMP2 shRNA vectors (termed KD#1 and KD#2), and the efficiency of EMP2 downregulation is shown in Fig. S1. Compared to the shCTRL, KD#1 reduced EMP2 by 31.4% while KD#2 reduced expression by 95% (Fig. S2). HIF1α protein expression was initially measured under normoxic conditions, and interestingly, EMP2 overexpression was sufficient to increase HIF1α protein expression even under normoxic conditions (Fig. S2A). In contrast, the WT, shCTRL, and KD cells demonstrated low to undetectable HIF1α protein expression in normoxia.

**Figure 5:**
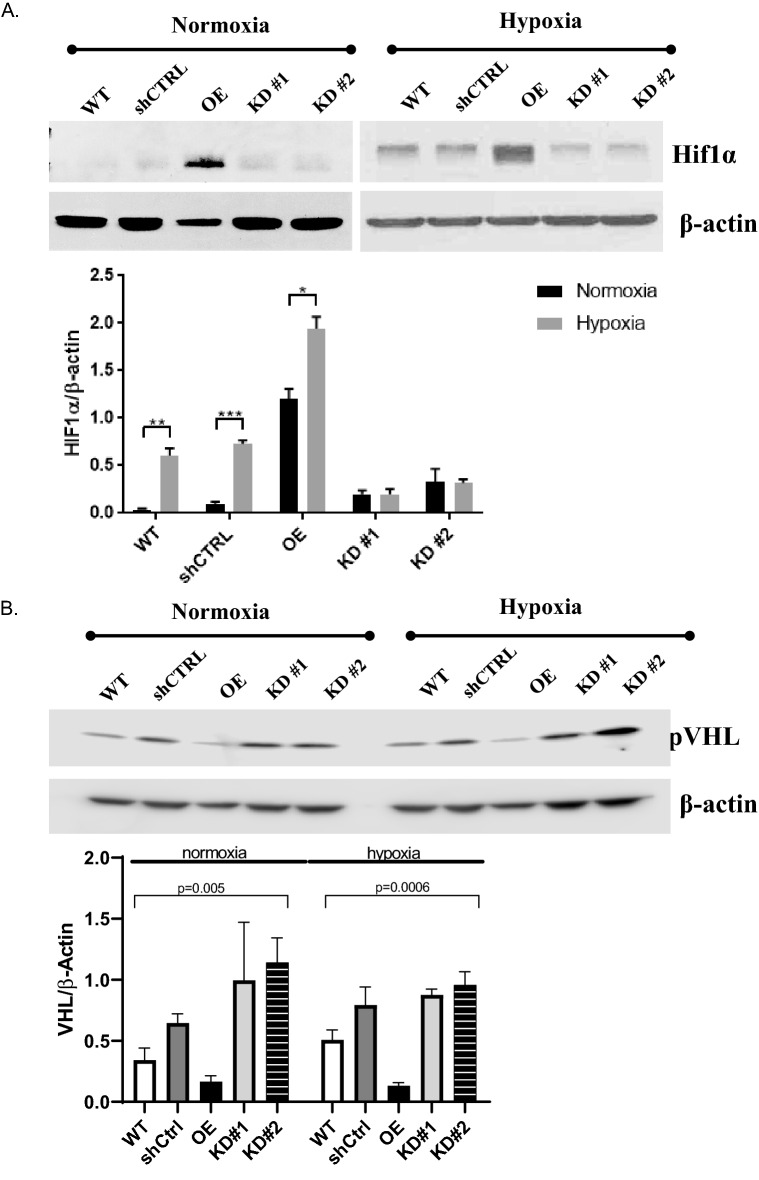
EMP2 expression modulates HIF1α and VEGF protein expression. (**A**, **B**) Analysis of HIF1α or VHL protein expression in WT, shRNA control (shCTRL), OE, shRNA KD #1, and shRNA KD#2 ARPE-19 cells under normoxic or hypoxic (0.5% O2 for 2–4 h) conditions, respectively. Quantification of protein expression under hypoxic conditions was determined using the LI-Cor Odyssey FC machine with images visualized using Image Studio software (Ver. 5.2). Expression of HIF1α or VHL was normalized to beta actin, for a minimum of 3 independent replicates. Bar diagrams depict mean values and standard error of the mean. Statistical significance was established using Student’s *t*-test (unpaired, two-tailed) for HIF1a or a one-way ANOVA for pVHL. Original, uncropped blots are presented in Supplementary Fig. S3.

Next, we measured the effect of hypoxia on these proteins. Under hypoxic conditions, increased HIF1α and VEGF protein expression were observed in the EMP2 overexpressing compared to WT and shCTRL cell lines (Fig. S2B). Conversely, significantly reduced HIF1α and VEGF protein expression were observed in both shRNA knock down lines, while the shRNA control line had expression levels similar to the unmanipulated WT ARPE-19 cell line. We next examined the relative induction of HIF1α expression under both normoxia and hypoxia in all the cell lines. Significantly, while HIF1α was induced under hypoxic conditions in WT, shCTRL, and OE, no induction of HIF1α protein expression occurred in response to hypoxia in both shRNA knock down lines (Fig. 5A), suggesting that EMP2 expression is necessary for HIF1α induction in RPE cells.

Adaptive cellular responses to hypoxia are orchestrated by oxygen sensing mechanisms. In particular, the von Hippel-Lindau tumor suppressor protein (pVHL) regulates HIF-mediated adaptation, a process essential for normal RPE and retinal vasculature maintenance^21^. In the presence of oxygen, via recruitment of the E3 ubiquitin complex, pVHL promotes proteasomal degradation of HIF1α^22^. Under hypoxic conditions, pVHL is downregulated, allowing Hif1α stabilization. To measure VHL levels, ARPE-19 cells with modified EMP2 levels were grown under hypoxic or normoxic conditions and pVHL levels determined using western blot analysis (Fig. 5B). pVHL levels inversely varied with EMP2 under both hypoxic and normoxic conditions, providing a novel link through which EMP2 regulates HIF1α.

### EMP2 expression regulates migration of HUVEC endothelial cells

**Figure 6:**
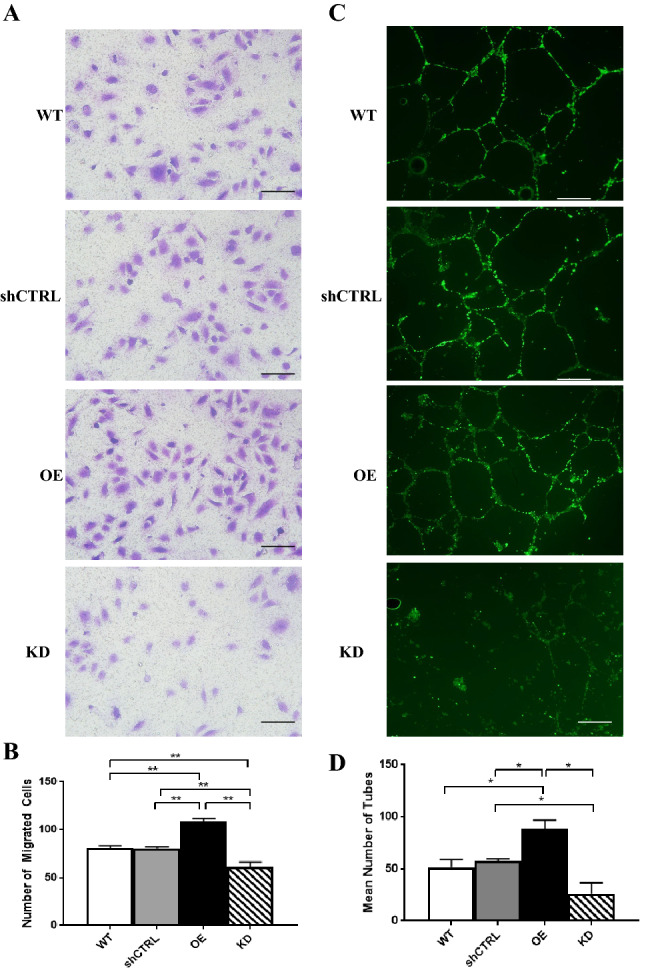
EMP2 expression regulates migration and tube formation of HUVEC endothelial cells. (**A**) Conditioned media was isolated from WT, shRNA control, OE, and shRNA KD#2 (labeled as KD) ARPE-19 cells exposed to 4 h of hypoxic stress (0.5% O2) and used in a transwell cell migration assay. Numbers of migrated cells were imaged and quantified (**B**). Three independent experiments were conducted, and statistical significance was established using Student’s *t*-test (unpaired, two-tailed). Bar diagrams depict mean values and standard error of the mean. Scale bar = 50 µm. (**C**) The morphology of HUVEC cells was analyzed following growth in WT, shRNA control, OE, and shRNA KD ARPE-19 conditioned media. Coverslips were stained with Calcein AM, imaged, and the number of tubes manually counted in a masked fashion. (**D**) Quantification of the number of tubes imaged above. Experiments were repeated three times, and statistical significance was established using an ANOVA test/Student’s *t*-test (unpaired, two-tailed). Bar diagrams depict mean values and standard error of the mean. Scale bar = 50 µm.

We next assessed whether EMP2 regulation results in paracrine secretion with functional responses in a HUVEC transwell migration assay. HUVEC cells were incubated with conditioned media from ARPE-19 cells with varying EMP2 expression that had been exposed to hypoxic stress, and migration through a transwell filter was quantified. Overexpression of EMP2 produced conditioned media that allowed for significantly increased numbers of cells migrated compared to WT (1.3-fold change, *p* = 0.0032) and shRNA control (1.3-fold change, *p* = 0.0022) cells (Fig. 6A,B). Conditioned media from cells with knockdown of EMP2 demonstrated reduced cell migration compared to media from WT (− 1.3-fold change, *p* = 0.0255) or shRNA control (− 1.3-fold change, *p* = 0.0241) cells (Fig. 6A,B).

### EMP2 expression regulates tube formation of HUVEC endothelial cells

Capillary tube formation of HUVEC endothelial cells was assessed as another functional measure of EMP2 expression in the ARPE-19 model. The mean number of capillary tubes formed following incubation in conditioned media from the ARPE-19 panel with varying EMP2 expression was quantified. Overexpression of EMP2 resulted in significantly increased tube formation compared to WT (1.7-fold change, *p* = 0.0317) and shRNA control (1.5-fold change, *p* = 0.0269) cells (Fig. 6C,D). Conversely, knock down of EMP2 produced fewer numbers of tubes and more disorganized morphology compared to shRNA control (− 2.2-fold change, *p* = 0.0458) cells (Fig. 6C,D).

## Discussion

In this study, we attempt to clarify the relationship between the tetraspan membrane protein EMP2 and various mediators of angiogenesis including HIF1α and VEGF under hypoxic conditions using a model RPE cell line. Using a novel panel of ARPE-19 cell lines modified to overexpress or reduce EMP2 protein, we demonstrate that there is a positive correlation between EMP2 expression and HIF1α and VEGF expression, at both the RNA and protein level, and an inverse correlation with pVHL. Additionally, we demonstrate that these EMP2 mediated changes under hypoxic conditions alter the secretion of a paracrine factor in conditioned media that can result in significant functional changes in both a HUVEC transwell migration assay as well as capillary tube formation assay.

HIF1α is considered a master transcriptional regulator of cellular and developmental responses to hypoxia, leading to the transcription of dozens of genes involved in angiogenesis, erythropoiesis, cell proliferation, and survival^23,24^. HIF1β is a constitutively expressed subunit, while HIF1α is an oxygen-regulated subunit. Under normoxic conditions, HIF1α is rapidly degraded via the ubiquitin mediated protease pathway, while protein degradation is prevented under hypoxia to allow HIF1α levels to accumulate rapidly^25^. HIF1α is typically expressed only under conditions of hypoxic stress; however, EMP2 overexpression alone under normoxia appears sufficient to induce HIF1α. Mechanistically, we propose that EMP2 mediated changes in HIF-1α occur through an inability to ubiquitylate HIF-1α due to loss of pVHL. This would result in HIF-1α stabilization, increased HIF transcriptional activity, and finally up-regulation of HIF target genes such as VEGF. EMP2 overexpression in the setting of hypoxia appears to have a greater than additive effect on HIF1α expression, and knock down of EMP2 greatly reduces HIF1α expression, even under strong hypoxic stress. While the exact link between EMP2 and pVHL is unknown, no significant differences in VHL mRNA levels were observed between cell lines (data not shown), suggesting a post-transcriptional regulation of VHL.

The RNA sequencing data and the eyeIntegration data are consistent in showing high levels of EMP2 in RPE and cornea. The RPE is important in abnormal retinal neovascularization, and the major sources of VEGF-A in the retina are known to include the RPE, Muller cells, and ganglion cells^26,27^. VEGF has been identified to be an extremely important mediator of retinal neovascularization^1^, with current therapies for pathologic neovascularization focused on anti-VEGF therapy^4^. The observation that EMP2 is a novel regulator of VEGF supports our hypothesis that EMP2 may serve as a novel target for treatment of pathologic neovascularization in the retina.

This study supports further investigation of EMP2 as a therapeutic target for retinal neovascularization in diseases such as age-related macular degeneration (AMD), retinopathy of prematurity, and diabetic retinopathy. Current therapy involves directly targeting VEGF, however there is evidence of a ceiling effect with real world anti-VEGF treatment^9,10,28^, and investigation of other therapeutic agents is needed. EMP2 may be a novel regulator of angiogenesis in RPE cells, and targeting of EMP2 may potentially allow control of VEGF expression through an alternate pathway, thereby bypassing some of the issues with direct anti-VEGF therapy. Our group has evidence that inhibition of EMP2 through antibody blockade is effective in other models of angiogenesis, including corneal neovascularization^13^ and proliferative vitreoretinopathy^20^ as well as animal tumor models of glioblastoma and breast cancer^19,26,27^. Further work with retinal and choroidal endothelial cells^29^, primary RPE cells, and in vivo models of retinal neovascularization should be pursued to evaluate the therapeutic benefit of targeting EMP2 in retinal vascular disease.

## Materials and methods

### Cell culture

Retinal pigment epithelium (ARPE-19) cells were obtained from the ATCC (catalog number CRL-2302) and cultured using Dulbecco’s modified Eagle’s media F-12 supplemented with 10% fetal bovine serum, 1% 100X HEPES, 1% L-glutamine, and 1% Penicillin/Streptomycin at 37 °C in a 5% CO_2_ humidified incubator (normoxia). EMP2-overexpressing (OE) and vector control (VC) ARPE-19 cell lines were created through stable transfection of ARPE-19 cells with an EMP2-overexpressing or a control construct via retroviral transduction, as previously described^23^. EMP2 reduction in ARPE-19 cells was facilitated through lentiviral delivery of short-hairpin RNA specific to EMP2 (Sigma-Aldrich, St. Louis, MO). Two different knockdown clones (KD #1, TRCN0000322386 and KD #2, TRCN0000322911) were generated and evaluated for efficiency. An additional control ARPE-19 cell line (shCTRL) was obtained through lentiviral infection of cells with a non-target short-hairpin RNA control construct. EMP2 expression in VC and shCTRL cell lines did not differ compared to ARPE-19 wildtype cells, as observed via western blot (Fig. S1).

ARPE-19 cell lines were cultured for two weeks in normoxic conditions prior to exposure to hypoxia. Hypoxic conditions were created by placing the cell cultures into a hypoxic chamber (BioSpherix, Parish, NY) at 37 °C with 0.5% O_2_, 5% CO_2_, and balance N_2_ (UCLA Cylinder Management, Los Angeles, CA). Based on previous evidence of maximum HIF1α induction occurring after 4 h of hypoxia, cell cultures were kept in the hypoxic chamber for 2–4 h^30^. Supernatants from cell cultures were collected 4 h post-hypoxia induction, centrifuged to remove cell debris, and stored at − 20 °C. The culture plate wells were rinsed with 1× DPBS (Corning, Oneonta, NY), and cell lysates were prepared immediately using Laemmli lysis buffer.

Human Umbilical Vein Endothelial Cells (HUVECs, ATCC, Manassas, VA) were cultured using VEC MCDB-131 complete media (VEC Technologies, Rensselaer, NY) at 37 °C in a humified 5% CO_2_ incubator. HUVECs used for in vitro assays were used at passage 4.

### SDS-PAGE/western blot analysis

ARPE-19 cell lysates were prepared using Laemmli lysis buffer. For EMP2 detection, lysates were treated with N-glycosidase F (New England Biolabs, Beverly, MA) for 90 min at 37 °C to remove N-link glycosylation. Proteins were separated on 4–20% SDS-PAGE gels (ThermoFisher Scientific, Grand Island, NY) under reducing conditions before transfer to nitrocellulose membranes. The membranes were blocked with 10% nonfat dry milk in tris-buffered saline with 0.1% Tween-20 (TBSt) and probed in 5% nonfat dry milk in TBSt, with the following antibodies: anti-human EMP2 antisera^11^ (1:2000 dilution), anti-HIF1α (1:500 dilution; clone 54, BD Bioscience, San Jose, CA), anti-VHL (1:1000, clone 6457, Cell Signaling), anti-VEGF (1:200 dilution; clone A-20, Santa Cruz Biotechnology, Dallas, TX), and anti-β-Actin (1:20,000 dilution; clone 19D59, US Biologicals, Salem, MA). Protein bands were detected using horseradish peroxidase (HRP)-labelled secondary antibodies (Southern Biotech, Birmingham, AL) and visualized with chemiluminescence using Crescendo Western Substrate (EMD Millipore, Burlington, MA). Bands were detected using via X-ray film or by the LI-Cor Odyssey Fc imaging System. Bands via the LI-Cor imaging system were processed using the Image Studio software with exposure times set between 30 s and 5 min (Ver. 5.2; LI-Cor, Lincoln, Nebraska).

Band density in both cases was quantified using NIH ImageJ software. Samples were normalized to β-Actin to account for loading variation. At least three individual experiments were performed. Data were assessed for normality using the Shapiro–Wilk test, and statistical significance established using Student’s *t*-test (unpaired, two-tailed).

### Cell migration assay

HUVECs were seeded into the top compartment of the transwell insert of Boyden chambers (24-well plate, Corning). 300 µl of conditioned media from WT, OE, KD, and shCTRL cells were added to the bottom compartment of the chambers. Chambers were placed in a humidified incubator with 5% CO_2_ at 37 °C for 4 h. The transwell inserts were gently washed with 1× DPBS (Corning), fixed with 4% paraformaldehyde, and stained with 0.2% crystal violet. The inserts were then washed with distilled water and left to dry overnight at room temperature. Lastly, the inserts were imaged using an Olympus BX-51 (Waltham, MA) microscope and migrated cells were quantified. Three independent experiments were conducted, and statistical significance was established using Student’s *t*-test (unpaired, two-tailed).

### Tube formation assay

Sterile glass cover slips were placed in 12-well plates, and 50 µl per cm^2^ of Geltrex (Thermofisher Scientific) was coated onto each cover slip’s surface. The plates were incubated for 30 min at 37 °C. HUVECs were seeded onto the coated coverslips with the previously collected conditioned ARPE-19 media. The coverslips were incubated for 6 h in a humidified incubator with 5% CO_2_ at 37 °C. Calcein AM (ThermoFisher Scientific) was added to each well and incubated for 30 min at 37 °C. The coverslips were carefully removed and mounted onto slides, evaluated, and quantified using a 10× objective on a fluorescent Olympus BX-51 (Waltham, MA) microscope. All fields were manually counted in a masked fashion for each culture condition. Each experiment was repeated three times, and statistical significance was established using an ANOVA test/Student’s *t*-test (unpaired, two-tailed).

### RNA sequencing

Total RNA was extracted from cells using RNeasy Mini Kit (Qiagen) and RNA concentration was measured using a Nanodrop spectrophotometer (ThermoFisher Scientific). Three replicates of each sample at 2 µg of RNA were submitted for sequencing and analysis (Technology Center for Genomics and Bioinformatics, UCLA). The methods for EyeIntegration RNA sequencing and library preparation was using the KAPA RNA HyperPrep Kit with RiboErase (Roche Sequencing, cat#KK8561, Pleasanton, CA, USA), according to the manufacturer’s instructions. The work flow consisted of rRNA depletion, cDNA generation, end repair to generate blunt ends, A-tailing, adaptor ligation, and PCR amplification. Different adaptors were used for multiplexing samples in one lane. Sequencing was performed on the Illumina HiSeq3000 System for a single-read 50 run (Illumina, San Diego, CA). Data quality check was done on Illumina SAV. Demultiplexing was performed with the Illumina Bcl2fastq2 v 2.17 program. The raw data has been deposited into the NCBI’s Gene Expression Omnibus (ascension number: GSE151610). The reads were mapped to the latest UCSC transcript set using STAR—2.27a and GRCh38. After obtaining gene counts, the counts were normalized by TMM. The Principal Component Analysis (PCA) was applied to the transcript counts. Differential gene expression analysis was performed using DEseq2 (version 1.26.0). Hypergeometric testing was performed by the clusterProfiler package (FDR < 0.05) using a background list of all genes tested for significant expression changes. For all results of differential gene expression analysis, the filter of FDR-adjusted *p* < 0.01 was applied. All figures were generated in R (RStudio, version 1.2.5033). Comparison of three condition levels (KD, WT, and OE) to obtain differentially expressed genes was performed using the likelihood ratio test (LRT) within DEseq2 (version 1.30.1). The top 1000 genes that passed the FDR < 0.01 filter value were input into clustering analysis. The degPatterns tool within the DEGreport R package (version 1.26.0) was used to identify and visualize gene groups with similar expression profiles using a hierarchical clustering approach. Default parameters were used except the minimum number of genes per cluster was set to 1 (minc = 1). Gene ontology enrichment analysis was performed with the clusterProfiler R package (version 3.18.1) on genes belonging to Groups 1 (n = 506) and 2 (n = 391). The background gene set for hypergeometric testing included all genes tested for significance in the LRT (gene with ≥ 10 counts across all samples). The hypergeometric test used Benjamini–Hochberg correction and an adjusted p cutoff of 0.05. Dot plots generated from GO analysis results included the top 10 enriched terms for the biological property ontology ordered by gene ratio, the number of genes related to a GO term divided by the total number of significant genes. Heatmaps were generated in R using pheatmap^31^, version 1.0.12 (https://CRAN.R-project.org/package=pheatmap). The chord diagram^32^ was created in “GOplot”, version 1.0.2 (https://CRAN.R-project.org/package=GOplot).

### EyeIntegration analysis

The NEI EyeIntegtration web resource (available in the public domain at https://eyeIntegration.nei.nih.gov), is an interactive web app that allows for transcript and gene comparisons across the largest set of transcriptomes from curated human eye tissues and dozens of other body tissues^28^. EyeIntegration (version 1.05) was queried for differential expression of EMP2 using the Gene 2019 dataset for relative expression in specific eye tissues of interest including adult cornea, adult retina, fetal retina, adult RPE, fetal RPE, and RPE cell line. Expression in lung was used as a positive control, and expression in whole blood as a negative control. Box plot visualizations were generated using the NEI EyeIntegration web app.

## Supplementary Information

Supplementary Information.

## Data Availability

The datasets used and/or analyzed during the current study available from the corresponding author on reasonable request. Uncropped images of the western blots used in this paper are provided in Supplementary Fig. S3.

## Abbreviations

AMD: Age related macular degeneration
ARPE-19: Adult retinal pigment epithelial cell line-19 (ATCC: #CRL-2302)
EMP2: Epithelial membrane protein 2
HIF1α: Hypoxia inducible factor 1-alpha
HUVEC: Human umbilical vein endothelial cells
KD: Knock down
OE: Overexpressing
RPE: Retinal pigment epithelium
VC: Vector control
VEGF: Vascular endothelial growth factor
WT: Wild type
