# Supplementary material for: Epithelial membrane protein 2 (EMP2) regulates hypoxia-induced angiogenesis in the adult retinal pigment epithelial cell lines
Source: Sci Rep. 2022 Nov 12;12:19432. doi: 10.1038/s41598-022-22696-x (PMC9653491; doi:10.1038/s41598-022-22696-x)
Supplement: Supplementary file 1 — Supplementary Information. [file 41598_2022_22696_MOESM1_ESM.pptx]

## Slide 1
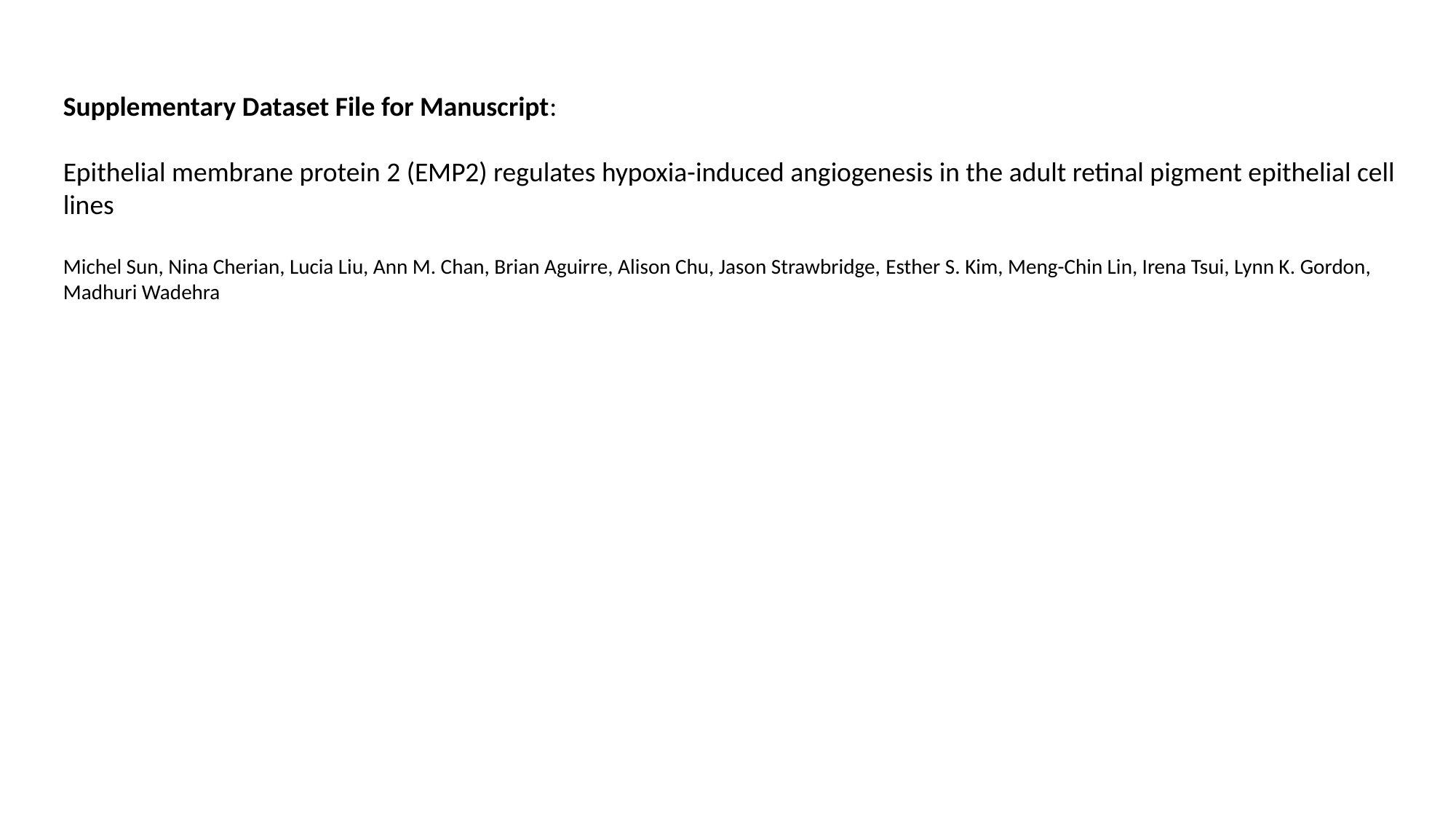

Supplementary Dataset File for Manuscript:
Epithelial membrane protein 2 (EMP2) regulates hypoxia-induced angiogenesis in the adult retinal pigment epithelial cell lines
Michel Sun, Nina Cherian, Lucia Liu, Ann M. Chan, Brian Aguirre, Alison Chu, Jason Strawbridge, Esther S. Kim, Meng-Chin Lin, Irena Tsui, Lynn K. Gordon, Madhuri Wadehra

## Slide 2
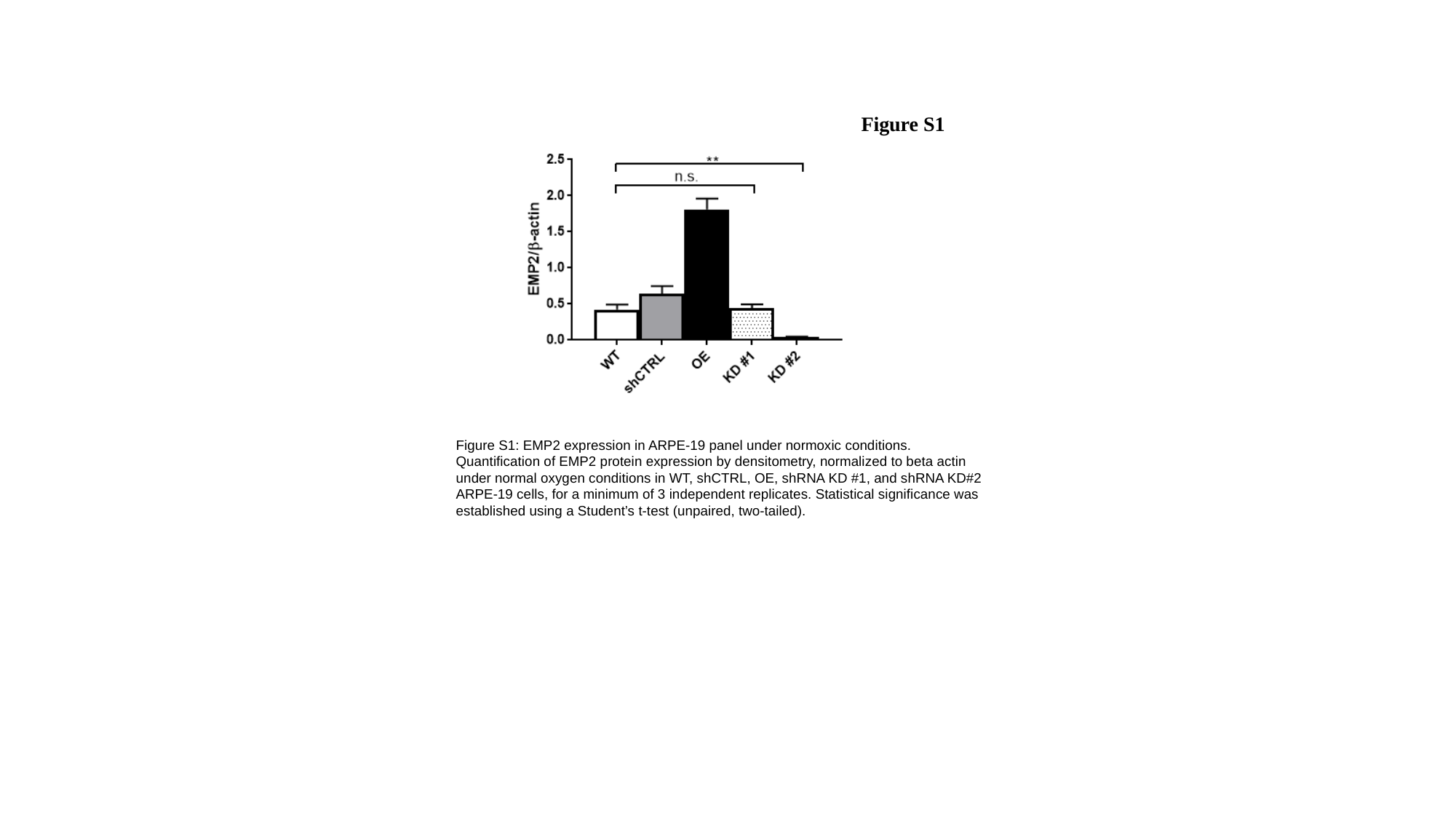

Figure S1
Figure S1: EMP2 expression in ARPE-19 panel under normoxic conditions. Quantification of EMP2 protein expression by densitometry, normalized to beta actin under normal oxygen conditions in WT, shCTRL, OE, shRNA KD #1, and shRNA KD#2 ARPE-19 cells, for a minimum of 3 independent replicates. Statistical significance was established using a Student’s t-test (unpaired, two-tailed).

## Slide 3
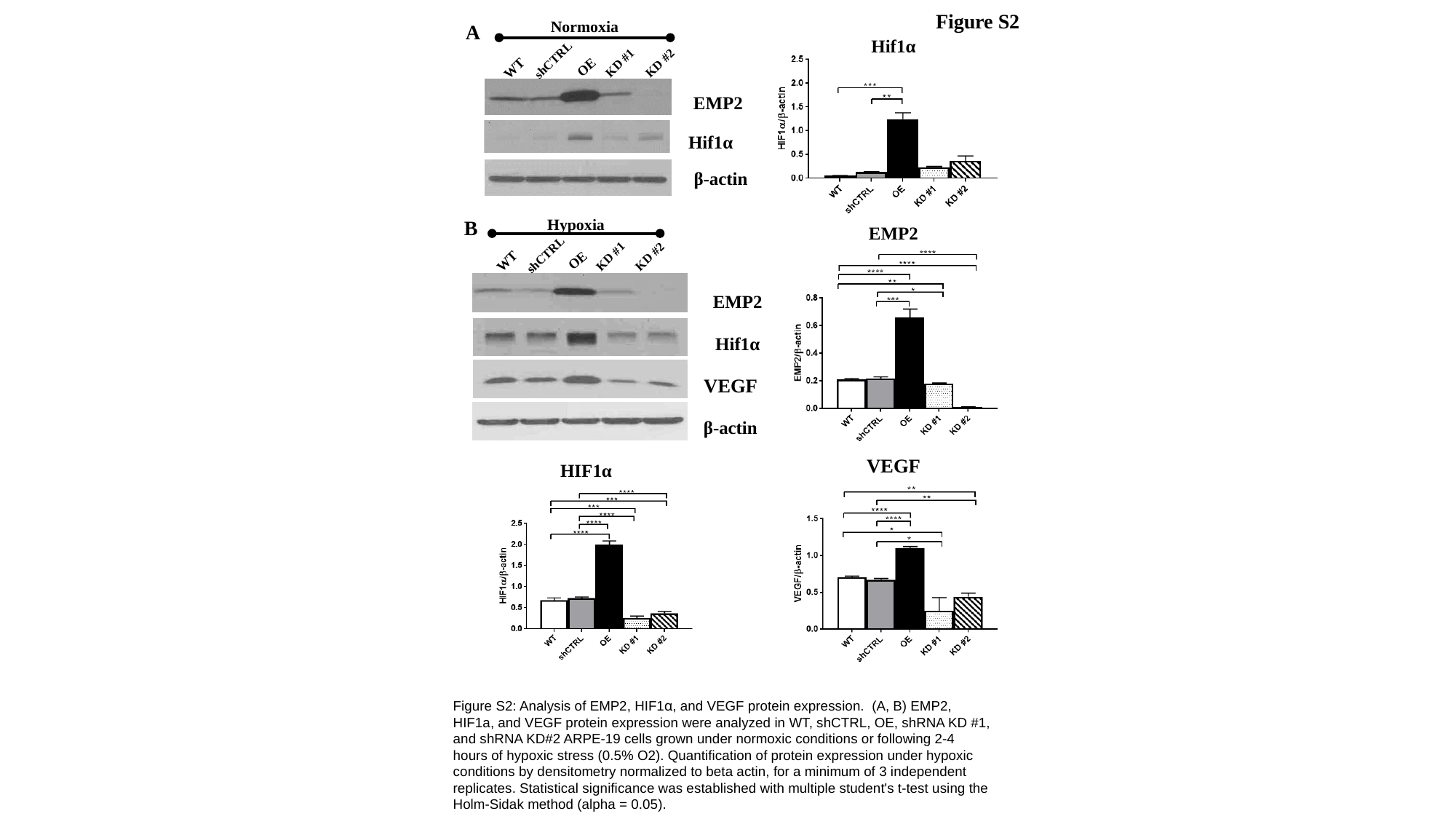

Figure S2
Normoxia
shCTRL
KD #1
KD #2
OE
WT
A
EMP2
Hif1α
β-actin
Hif1α
Hypoxia
shCTRL
KD #1
KD #2
OE
WT
EMP2
Hif1α
VEGF
β-actin
B
EMP2
VEGF
HIF1α
Figure S2: Analysis of EMP2, HIF1α, and VEGF protein expression. (A, B) EMP2, HIF1a, and VEGF protein expression were analyzed in WT, shCTRL, OE, shRNA KD #1, and shRNA KD#2 ARPE-19 cells grown under normoxic conditions or following 2-4 hours of hypoxic stress (0.5% O2). Quantification of protein expression under hypoxic conditions by densitometry normalized to beta actin, for a minimum of 3 independent replicates. Statistical significance was established with multiple student's t-test using the Holm-Sidak method (alpha = 0.05).

## Slide 4
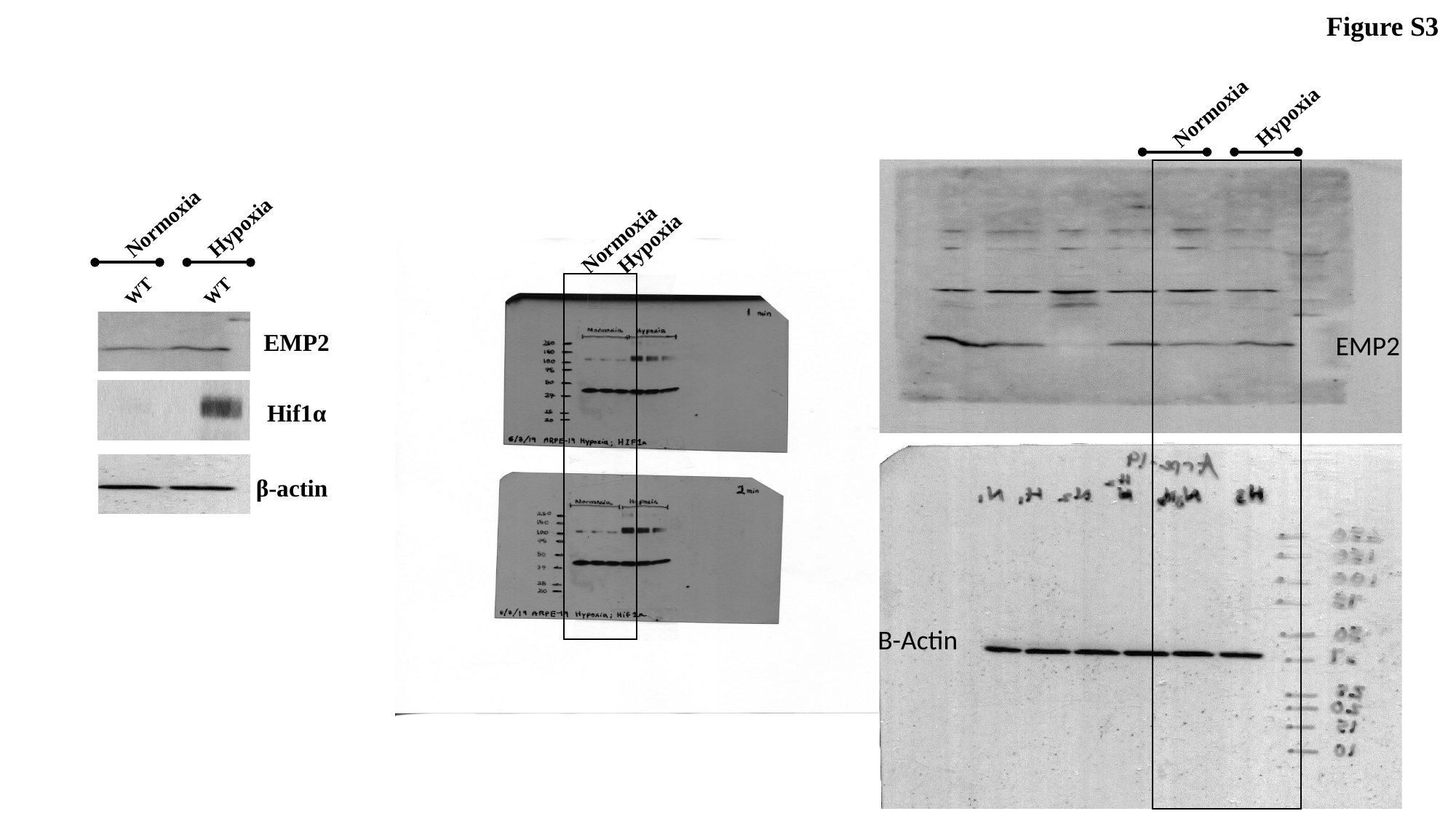

Figure S3
Normoxia
Hypoxia
Normoxia
Hypoxia
Normoxia
Hypoxia
WT
WT
EMP2
EMP2
Hif1α
β-actin
B-Actin

## Slide 5
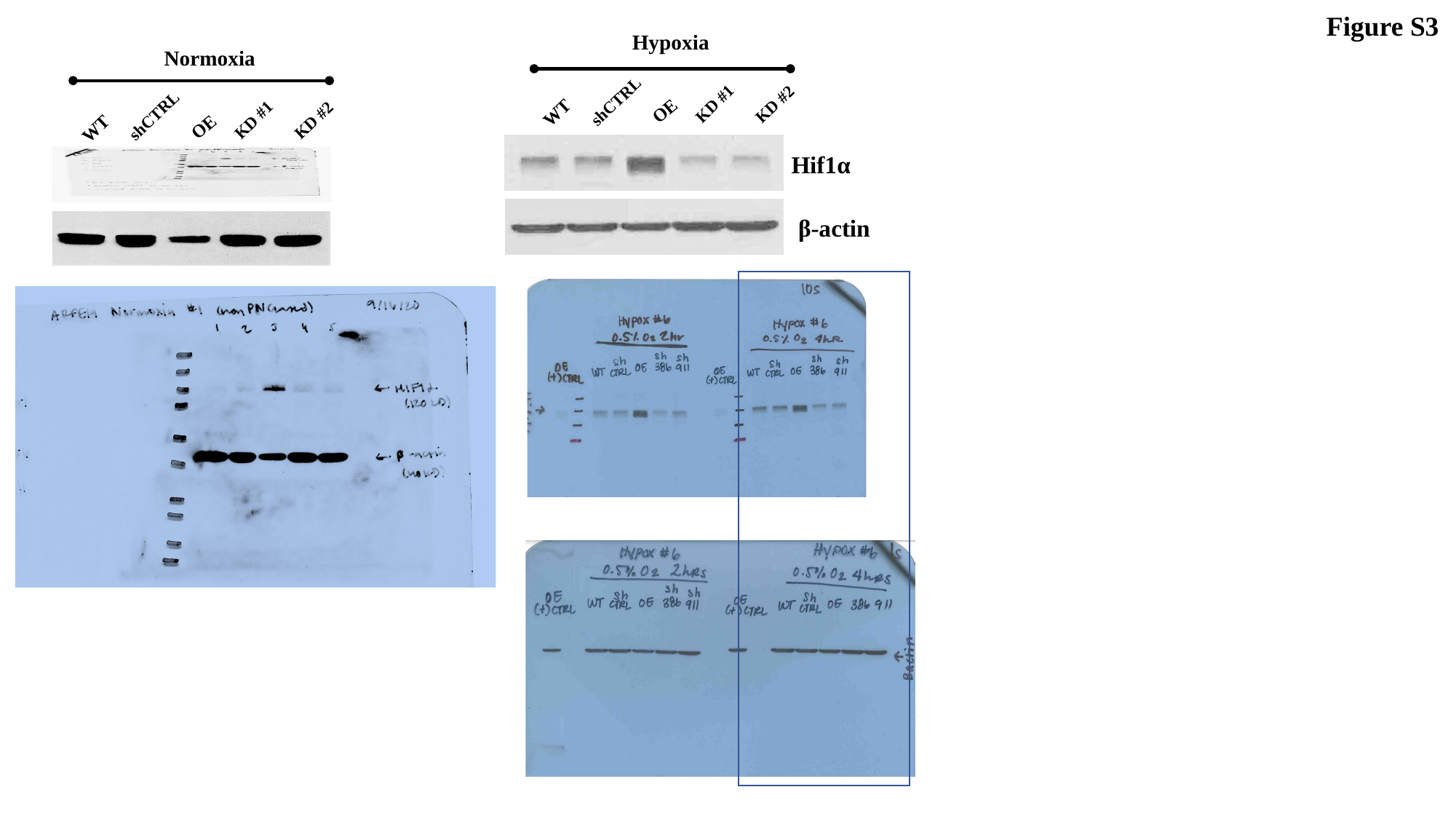

Figure S3
Hypoxia
Normoxia
shCTRL
KD #1
KD #2
OE
WT
shCTRL
KD #1
KD #2
OE
WT
Hif1α
β-actin

## Slide 6
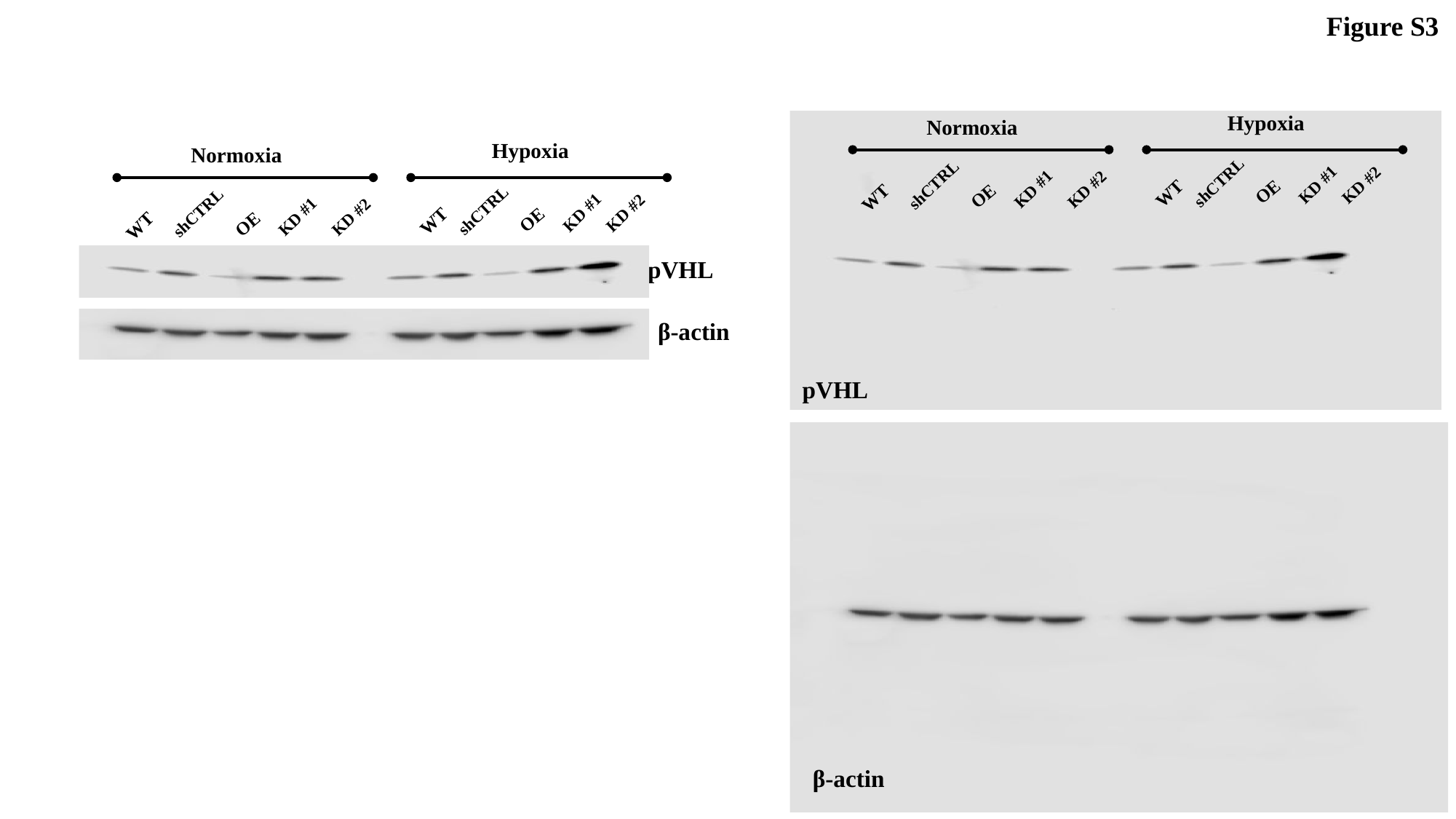

Figure S3
Hypoxia
Normoxia
Hypoxia
Normoxia
shCTRL
KD #1
KD #2
shCTRL
KD #1
KD #2
OE
WT
OE
WT
shCTRL
KD #1
KD #2
shCTRL
KD #1
KD #2
OE
WT
OE
WT
pVHL
β-actin
pVHL
β-actin

## Slide 7
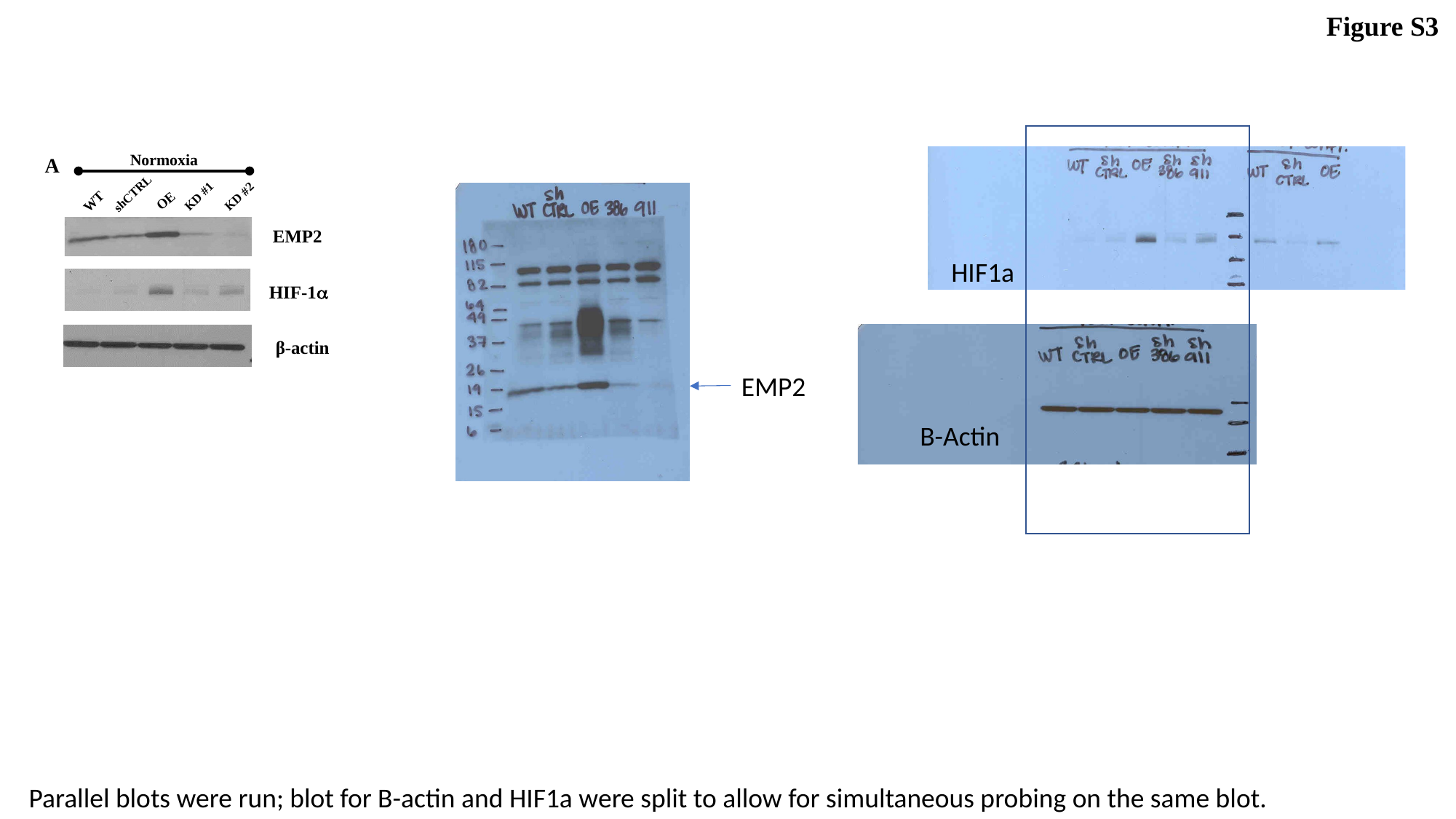

Figure S3
Normoxia
shCTRL
KD #1
KD #2
OE
WT
A
EMP2
HIF1a
HIF-1a
β-actin
EMP2
B-Actin
Parallel blots were run; blot for B-actin and HIF1a were split to allow for simultaneous probing on the same blot.

## Slide 8
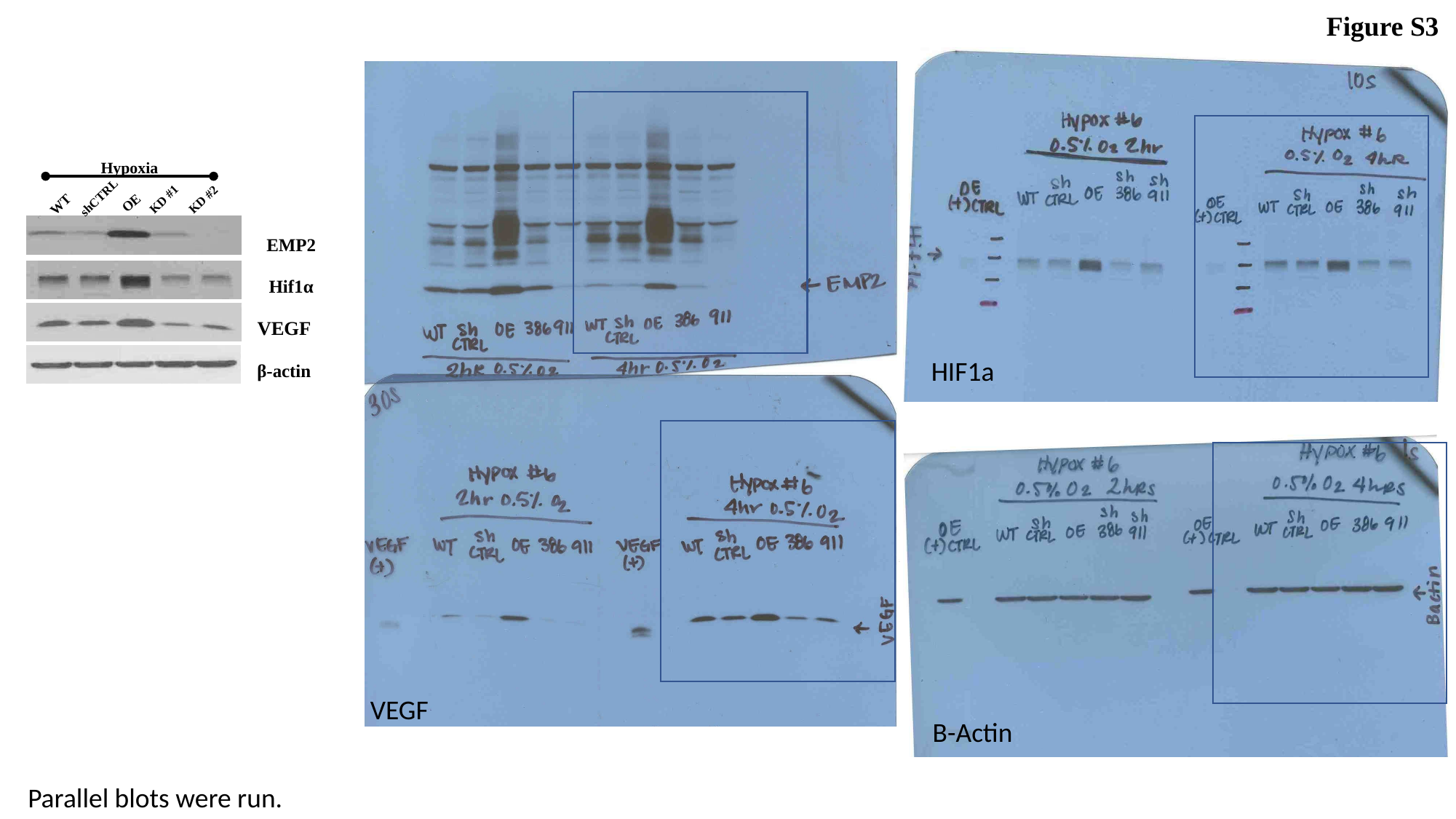

Figure S3
Hypoxia
shCTRL
KD #1
KD #2
OE
WT
EMP2
Hif1α
VEGF
β-actin
HIF1a
VEGF
B-Actin
Parallel blots were run.
